# Supplementary material for: Disease-modifying therapies and features linked to treatment response in type 1 diabetes prevention: a systematic review
Source: Commun Med (Lond). 2023 Oct 5;3:130. doi: 10.1038/s43856-023-00357-y (PMC10550983; doi:10.1038/s43856-023-00357-y)
Supplement: Supplementary file 1 — Supplementary Information [file 43856_2023_357_MOESM1_ESM.pdf]

Supplemental Note 1: PubMed/Embase Search Strategy

#1 Latent Autoimmune Diabetes in Adults [Mesh] OR "Diabetes Mellitus, Type 1 "[Mesh] OR "diabetes mellitus, type 1/prevention and control"[MeSH Terms] 81438 records

#2 ((((prevent* [Title/Abstract] OR delay [Title/Abstract] OR immunomod* [Title/Abstract] OR immune therapy [Title/Abstract] OR "disease modifying therapy" [Title/Abstract] OR Antibodies, Monoclonal, Humanized / therapeutic use* [Title/Abstract] OR C-peptide* [Title/Abstract]) OR ("autoantibodies/analysis"[MeSH Terms]))

1922455 records

#3 (randomized controlled trial[Publication Type] OR randomized controlled trial[Title/Abstract]) OR (random*[Title/Abstract] OR rct[Title/Abstract] OR randomized[Title/Abstract])
1422392 records

#4 #1 AND #2 13273 records

#5 #4 AND #3 1169 records

Filters: Human, English, 1996-present 880 records

Supplementary Table 1. Studies in individuals with new or recent onset T1D (<1 year since diagnosis)

Study ID	n	Intervention	Multi-center	Blinding	Primary Outcome(s)	Age range (years)	Positive ?
Ergun-Longmire 2004 [1]	197	1 mg po insulin vs. 10 mg po insulin vs. placebo x 6-36 mo	Yes	Double	β Cell function	5-60	No
Enander 2018 [2]	54	48-72 hrs IV insulin vs. usual care	Yes	None	Not specified	2-17.99	No
Chaillous 2000 (Diabète Insuline Orale) [3]	131	2.5 mg po human biogenetic insulin daily vs. 7.5 mg po insulin vs. placebo x 12 mo	Yes	Double	β Cell function	7-40	No
Pozzilli 2000 [4] (IMDIAB VII)	80	5 mg daily po insulin x 12 mo vs. placebo	Yes	Double	HbA1c; Insulin dose; β Cell function	6-22	No
Crinò 2004 [5] (IMDIAB IX)	64	25 mg/kg/d nicotinamide + 15 mg/kg/d vitamin E vs. 25 mg/kg/d nicotinamide x 2 yrs	Yes	Not Stated	β Cell function	4-18	No
Pitocco 2006 [6] (IMDIAB XI)	70	Calcitriol vs. Nicotinamide	Yes	None	β Cell function	6-22	No
Pozzilli 1997 [7] (IMDIAB IV)	84	15 mg/kg vitamin E vs. 25 mg/kg nicotinamide x 1 yr	Yes	Double	β Cell function	5-35	No
Coutant 1998 [8]	63	2.5mg/d linomide vs. placebo x 12 mo	No	Double	Not specified	10-20	n/a
Allen 1999 [9]	94	23106 colony forming units TICE BCG intradermal injection x 1 vs. placebo	Yes	Double	Incidence of remission	5-18	No
Keymeulen 2005 [10]<br>• Keymeulen 2010 [11] (follow-up)<br>• Demeester 2015 [12] (precision)	80<br>• 73<br>• 80	0.22 IU/kg/day ChAglyCD3 (otelixizumab) IV x 6 days vs. placebo	Yes	Double	β Cell function	12-39	Yes<br>Yes
Sherry 2011 [13] (Protégé)<br>• Hagopian 2013 [14] (follow-up)	516<br>• 462	14 d full dose, 14 d low dose, or 6 d full dose IV teplizumab vs. placebo at wk 0 and wk 26<br>• 24 mo follow-up	Yes	Double	HbA1c; Insulin dose<br>β Cell function	12-39	No<br>Yes
Herold 2013 [15]	63	14 d IV teplizumab vs. placebo	Yes	Double	β Cell function	12-39	Yes

Herold 2013 [16] (AbATE)<br>• Long 2016 [17] (precision)<br>• Long 2017 [18] (precision)	83<br>• 74<br>• 41	14 d IV teplizumab, with repeat at 1 yr vs. placebo	Yes	None; Lab blinded	β Cell function	8-35	Yes
Aronson 2014 [19] (DEFEND-1)	272	8 d IV otelixizumab (3.1 mg) vs. placebo, outcome at 12 months	Yes	Double	β Cell function	12-45	No
Ambery 2014 [20] (DEFEND-2)	179	8 d course IV otelixizumab (3.1 mg) vs. placebo	Yes	Double	β Cell function	12-45	No
Pescovitz 2009 [21]<br>• Pescovitz 2014 [22] (follow-up)<br>• Herold 2011 [23] (precision)<br>• Linsley 2019 [24] (precision)	87<br>• 77<br>• 78<br>• 54	IV rituximab days 1, 8, 15, and 22 vs. placebo	Yes	Double	β Cell function<br>β Cell function at 24 months	8-45	Yes<br>• No
Ortqvist 2004 [25]	56	5-7.5 mg/kg/d po diazoxide vs. placebo x 24 mo	Yes	Double	β Cell function	7-17	Yes
Walter 2009 [26]	188	0.1 mg sc inj of altered peptide ligand NBI-6024 at baseline, wk 2 and 4, and then qmo vs. placebo x 24 mo	Yes	Double	β Cell function	12-35	No
Rother 2009 [27]	128	5,000 units/d or 30,0000 units/d po human recombinant IFN-alpha vs. placebo x 1 yr	Yes	Double	β Cell function	3-25	Yes
Gottlieb 2010 [28]	126	600mg/m2/d po mycophenolate mofetil (MMF) x 2 yrs, vs. MMF+1m/kg IV Daclizumab x 2, vs. placebo	Yes	Double	β Cell function	8-45	No
Wherrett 2011 [29]	145	20μg sc inj GAD-alum x 3 vs. GAD-alum x 2 /alum x 1 vs. alum x 3	Yes	Double	β Cell function	3-45	No
Ludvigsson 2012 [30]	334	20ug sc inj GAD-alum x 4,vs. 20ug sc GAD-alum x 2/placebo x 2 vs. placebo x 4	Yes	Double	β Cell function	10-20	No
Ludvigsson 2020 [31] (DIABGAD)	64	450 d Vit. D + 90 d Ibuprofen + 20 μg sc inj GAD-alum x 2, vs. 40 μg GAD-alum, vs. Vit D +placebo+20 μg GAD-alum, vs. placebo	Yes	Double	Not specified	10-17.99	No

Ludvigsson 2021 [32] (DIAGNODE-2)	109	4mg GAD-alum intralymphatic injection monthly x 3 + oral vitamin D (2,000 IE daily for 120 days) vs. placebo	Yes	Double	β Cell function	6-45	No
Orban 2011 [33]<br>• Orban 2014 [34] (follow-up)<br>• Orban 2014 [35] (precision)<br>• Cabrera 2018 [36] (precision)<br>• Linsley 2019 [37] (precision)<br>• Eichmann 2020 [38] (precision)	112<br>• 112<br>• 87<br>• 74<br>• 105<br>• 59	10 mg/kg IV abatacept x 27 over two years vs. placebo<br>• 36 mo extended f/u (1 yr post tx cessation)	Yes	Double	β Cell function	6-45	Yes
Martin 2011 [39] (DIATOR)<br>• Strom 2012 [40] (precision)	89<br>• 89	80 mg/d atorvastatin vs. placebo x 18 mo	Yes	Double	β Cell function	18-39	No
Moran 2013 [41]	69	2mg/kg sc injection monthly canakinumab x 12 mo vs. placebo	Yes	Double	β Cell function	6-45	No
Moran 2013 [41] (AIDA)	69	100 mg daily sc inj anakinra x 9 months vs. placebo	Yes	Double	β Cell function	6-45	No
Gitelman 2013 [42] (START)<br>• Gitelman 2016 [43] (follow-up)	58<br>• 58	6.5 mg/kg IV ATG vs. placebo<br>24-mo follow up	Yes	Double blinded after 3 mo	β Cell function	12-35	No<br>No
Ataie-Jafari 2013 [44]	61	0.25mcg- 0.5 mcg/d po Alfacalcidol vs. placebo x 6 mo	Yes	Single	β Cell function; insulin dose	8-15	Yes
Nafei 2017 [45]	75	2000IU/day Vit. D3 vs. usual care	No	None	Not specified	4-12	Yes
Buckingham 2013 [46]	68	Hybrid closed loop using the Medtronic MiniMed system for 72-96 hrs vs. usual care	Yes	Outcomes masked	Not specified	6-<46	No
Griffin 2014 [47] (REPAIR-T1D)	68	50-100 mg po sitagliptin + 30-60 mg po lansoprazole x12 months vs. placebo	Yes	Double	β Cell function	11-36	No

Pozzilli 2020 [48]	67	Albiglutide sc injection 30 -50 mg weekly x 52 wks vs. placebo	Yes	Double	β Cell function	18-30	No
Haller 2018 [49]<br><br>• Haller 2019 [50] (follow-up)	89<br><br>• 89	2.5 mg/kg IV ATG vs. ATG + 6mg sc inj pegylated GCSF q2wks x 6 vs. placebo<br><br>• 24 mo extended f/u	Yes	Double	β Cell function	12-45	Yes<br><br>Yes
Lebenthal 2019 [51]	70	60 mg/kg IV Alpha-1 Antitrypsin (Glassia) x 22 (52 wks), vs. 120 mg/kg x 22 vs. placebo	Yes	Double	β Cell function	8-25	No
Quattrin 2020 [52] (T1GER)	84	Golimumab 0, 2 wk induction then q2wk maintenance inj vs. placebo x 52 wks	Yes	Double	β Cell function	6-21	Yes
vonHerrath 2021 [53]	308	12 mg/kg monoclonal anti-IL-21 antibody q6 weeks vs. daily liraglutide sc inj vs. anti-IL-21 + liraglutide vs. placebo x 54 wks	Yes	Double	β Cell function	18-45	Yes
Lagarde 2021 [54]	76	90 or 180 mg/kg IV human-derived alpha1-proteinase inhibitor wkly x 13 wks vs. 26 wks vs. placebo	Yes	Partial blinding	β Cell function	6-35	No
Kumar 2021 [55]	96	3 mo high dose po multi-strain probiotic vs. placebo	No	Double	HbA1c	2-12	Yes
Groele 2021 [56]	96	10^9 colony-forming units/day po L. rhamnosus GG and B. lactis Bb12 vs. placebo x 6 mo	n/a	Double	β Cell function	8-17	No
Gitelman 2021 [57]	67	400 mg po daily imatinib mesylate x 26 weeks vs. placebo	Yes	Double	β Cell function	18-45	Yes
Greenbaum 2021 [58] (EXTEND)	163	8 mg/kg IV Tocilizumab monthly x 7 vs. placebo	Yes	Double	β Cell function	6-17	No
Diggins 2021 (T1DAL) [59] (precision)	26/49	15 mg alefacept IM qwk x 12; 12 wk off, qwk x 12 vs. placebo	Yes	Double	β Cell function	12-35	No
Christie 2002 [60] (Precision)	97/188	Cyclosporin vs. placebo x12 mo	Yes	Double	n/a	10-35	n/a

Follow-up or precision studies describing a randomized trial that is already included in the table are listed as bulleted subheadings. Po – per oral/orally, IV – intravenous, IMDIAB –nicotinamide in recent-onset IDDM study , HbA1c - hemoglobin A1c, TICE BCG – Tice Bacillus Calmette-Guerin, ChAglyCD3 – Otelexizumab, AbATE - Autoimmunity-Blocking Antibody for Tolerance trial, DEFEND - Durable Response Therapy Evaluation for Early or New-Onset Type 1 Diabetes, Sc – subcutaneous, Inj – injection, Qmo – every

month, MMF – Mycophenolate Mofetil, GAD-Alum – Glutamic acid decarboxylase – alum, , f/u – follow-up, tx – treatment, DIATOR – Diabetes Intervention with Atorvastatin, AIDA – Anti-Interleukin-1 in Diabetes Action, START - Study of Antithymocyte Globulin for Treatment of New-onset T1DM, ATG – Antithymocyte Globulin, GCSF - Granulocyte Colony-Stimulating Factor, Q2wks – every 2 weeks

T1GER - SIMPONI to Arrest β-cell Loss in Type 1 Diabetes, Q6 – every 6, n/a – not applicable, EXTEND - Tocilizumab (TCZ) in New-onset Type 1 Diabetes, T1DAL - Inducing Remission in Type 1 Diabetes With Alefacept, IM – intramuscular, Qwk – every week

Supplementary Table 2. Metabolic outcomes from studies in individuals with new or recent onset T1D.

Study ID	n	Intervention	C-peptide AUC Analysis	Results (p value vs. control) (units in nmol/L or pmol/mL unless stated)
Enander [2] 2018	54	IV insulin vs. usual care	Mean ± SD of 2 hr MMTT AUC	Usual Care: 23.9 ± 40.6 nmol/L*min<br>IV insulin: 18.26 ± 16.45 (ns)
Sherry 2011 [13](Protégé)*<br><br><br><br>Hagopian 2013 [14](f/u)*	516<br><br><br><br>516	14 d full dose x 2, 14 d low dose x 2, or 6 d full dose x 2 IV teplizumab vs. placebo	1 yr median (IQR) change from baseline in 4 hr MMTT AUC<br><br><br><br>2 yr mean change from baseline in 4-hr MMTT AUC adjusted for age group and baseline value	Placebo: −0·14 (−0·30, 0·02) nmol/L*min<br>14 d full dose: -0.06 (-0.25, 0.12), p =0.0486;<br>14 d low dose: −0·13 (−0·33, 0·01);<br>6 d full dose: -0.08 (-0.31, 0.11)<br>Placebo:-0.191;<br>14 d full dose: -0.136, p=0.027;<br>14 d low dose: -0.198, p=0.968;<br>6 d full dose: -0.174, p=0.312
Herold 2013*[15]	63	IV teplizumab vs. placebo	12 mo mean (95% CI) 4 hr MMTT AUC	Placebo: 0.37 (0.32, 0.42);<br>Teplizumab: 0.45 (0.40, 0.51), p=0.03
Herold 2013 [16](AbATE)*	83	IV teplizumab, with repeat course at 1 yr vs. placebo	24 mo mean (95% CI) change in 4 hr MMTT ln(AUC + 1) adjusted for baseline value	Placebo: -0.46 (-0.57, -0.35);<br>Teplizumab: -0.28 (-0.36,-0.20), p=0.002
Aronson 2014 (DEFEND-1) [19]	272	IV otelixizumab vs. placebo	12 mo change in 2 hr MMTT AUC	Placebo: -0.2 ± 0.037;<br>Otelixizumab: 0.025 ± 0.025 p=0.58
Ambery 2014 (DEFEND-2)[20]	179	IV otelixizumab vs. placebo	Difference in 12 mo change in 2 hr MMTT adjusted for age, continent, and baseline value	–0.09 (95% CI –0.17 to 0; P = 0.051)
Pescovitz [21]2009*<br><br><br>Pescovitz 2014 [22](F/u)	87<br><br><br>77	IV rituximab vs. placebo	12 mo mean (95% CI) 2 hr MMTT $\log_e$([mean AUC]+1) adjusted for age and sex.<br><br>24 mo mean (95% CI) 2 hr MMTT $\log_e$([mean AUC]+1) adjusted for age and sex.	Placebo: 0.47 (0.39, 0.55)<br>Rituximab: 0.56 (0.50, 0.63), p=0.009<br><br><br>Placebo: 0.336 (0.245, 0.433)<br>Rituximab: 0.398(0.326, 0.473), p=0.15
Walter 2009[26]	188	NBI-6024 vs. placebo	24 mo mean ± SD 2 hr MMTT AUC	Placebo: 50 ± 45 pmol x min/ml;<br>NBI-6024: 57 ± 71; p=0.5

Rother 2009*[27]	128	Human recombinant interferon-α(hrIFN- α) vs. placebo	Mean ± SD % 2 hr MMTT AUC loss from 0-12 months	Placebo: 56±29 %;<br>5000 units hrIFN-α: 29±54, p=0.017;<br>30,000 units hrIFN-α: 48±35, p=0.599
Gottlieb 2010 [28]	126	Mycophenolate mofetil (MMF), vs. MMF+ Daclizumab (DZB), vs. placebo	2 yr geometric mean (95%CI) 2 hr MMTT AUC	Placebo: 0.27 (0.18 ,0.37);<br>MMF: 0.25 (0.14 , 0.37) p=0.41;<br>MMF +DZB: 0.28 (0.19 , 0.37) p=0.47
Wherrett 2011 [29]	145	GAD-alum x 3 vs. GAD-alum x2/ alum x 1 vs. alum x 3	Ratio (95% CI) of population mean from first 2 hr AUC of 4 hr MMTT adjusted for age, sex, and baseline C-peptide	GAD-alum ×3: 0.998 (0.779, 1.22), p = 0.98;<br>GAD-alum ×2/alum ×1: 0.926 (0.720, 1.13), p = 0.50)
Ludvigsson 2012 [30]	334	GAD-alum x 4,vs. GAD-alum x 2 placebo x 2 vs. placebo	15 mo. mean estimated treatment ratio (95%CI) of change in 2 hr MMTT C-peptide AUC.	GAD x 4: 1.18 (0.955 - 1.458) p=0.13;<br>GADx2/Placebo x 2: 1.149 (0.929 - 1.421), p=0.2
Ludvigsson 2021 (DIAGNODE-2)[32]	109	Intralymphatic GAD-alum + vitamin D (vs. placebo	Mean (95%CI) treatment effect ratio from 2 hr MMTT AUC	1.091 (0.845-1.408); p=0.5009
Orban 2011*[33]<br><br>Orban 2014 [34](f/u)*	112<br><br>112	Abatacept vs. placebo	2 year geometric mean (95%CI) of 2 hr MMTT AUC adjusted for age, sex, and baseline value<br><br>36 mo population mean (95%CI) MMTT 2-h AUC, adjusted for age, sex, and baseline value	Placebo: 0.266 (0.171, 0.368)<br>Abatacept: 0·375 (0.290, 0.465) (p=0.0029)<br><br>Placebo: 0.141 (0.071,0.215);<br>Abatacept: 0.217 (0.168,0.268), p=0.046
Moran 2013[41]	69	Canakinumab vs. placebo	12 mo mean (95%CI) difference in 2 hr MMTT AUC vs. placebo	0.01 (−0.11 to 0.14), p=0.86)
Moran 2013 [41](AIDA)	69	Anakinra vs. placebo	9 mo mean (95%CI) difference in 2 hr MMTT AUC vs. placebo	0.02 (−0.09 to 0.15), p=0.71
Gitelman 2013 [42](START)<br>Gitelman [43]2016 (f/u)	58<br><br>58	ATG vs. placebo	12 mo mean change (95%CI) in 2 hr MMTT AUC<br><br>24 mo change in the mean (95% CI) 2 hr C-peptide AUC from 4 hr MMTT, adjusted for baseline value	Placebo: −0.239 (−0.361,−0.118)<br>ATG: −0.195 (−0.292, −0.098), p=0.591<br><br>Placebo: -0.32 (-0.473,0.174);<br>ATG: -0.27 (-0.373,0.171), p=0.38

Buckingham 2013[46]	68	Hybrid closed loop (HCL) vs. usual care	Geometric mean (95%CI) 2 hr MMTT AUC	Usual care: 0.52 (0.32,0.75);<br>HCL: 0.43 (0.34,0.52) p=0.49
Griffin 2014 (REPAIR-T1D)[47]	68	Sitagliptin + lansoprazole vs. placebo	12 mo mean change (95%CI) in 2 hr MMTT AUC	Placebo: −253 (−383,−123) ;<br>Sitagliptin+Lansoprazole: −229 (−316,−142), p=0.77
Pozzilli 2020[48]	67	Albiglutide sc injection 30 -50 mg weekly x 52 wks vs. placebo	52 wk Difference in least squares means (95%CI) vs placebo for change in 2 hr MMTT AUC	0.04 (−0.13, 0.20) , p=0.6505
Haller 2018*[49]	89	Low-dose ATG vs. ATG + GCSF vs. placebo	12 mo geometric-like means (95%CI) 2 hr AUC of 4 hr MMTT adjusted for for sex, age, and baseline value.	Placebo: 0.406 (0.324, 0.494)<br>ATG: 0.646 (0.547, 0.750), p = 0.0003<br>ATG/GCSF: 0.528 (0.435, 0.627), p = 0.031)
Haller 2019 (f/u)*[50]	89		24 mo geometric-like means (95%CI) 2 hr AUC of 4 hr MMTT adjusted for for sex, age, and baseline value.	Placebo: 0.253 (0.177, 0.334)<br>ATG: 0.5 (0.412, 0.594) p=0<0.001<br>ATG+GCSF: 0.36 (0.281, 0.445)p=0.032
Lebenthal 2019[51]	70	60 mg/kg IV Alpha-1 Antitrypsin (A1AT) x vs. 120 mg/kg x 22 vs. placebo	52 wk change in 2 hr MMTT AUC	Placebo: -0.34<br>60mg/kg A1AT:- 0.55 p=0.677<br>120mg/kg A1AT:- 0.29 p=0.822
Quattrin 2020 (T1GER)*[52]	84	Golimumab vs. placebo	Mean ± SD 52 wk 4 hr MMTT AUC	Placebo: 0.43±0.39;<br>Golimumab: 0.64±0.42; p<0.001
vonHerrath 2021*[53]	308	Anti-IL-21 vs. liraglutide vs. combination vs. placebo	54 wk estimated mean (95%CI) treatment ratio based on change in 4 hr MMTT AUC	IL-21: 1.23 (0.97–1.57), p=0·093<br>Liraglutide: 1.12 (0.87–1.42), p=0.38<br>Combination: 1.48 (1.16–1.89), p=0.0017
Groele 2021[56]	96	L. rhamnosus GG and B. lactis Bb12 vs. placebo	6 mo median (IQR) 2 hr MMTT AUC	Placebo: 3.30 (2.14; 4.56) ng/mL<br>Treatment: 3.38 (2.24; 4.52), p=0.993
Gitelman 2021*[57]	67	Imatinib mesylate x 26 weeks vs. placebo	Mean difference (90% CI) 2 hr AUC from 4 hr MMTT adjusted for sex, baseline age, and baseline value	0.095 (–0.003 to 0.191), p=0.048
Greenbaum 2021 [58](EXTEND)	81 pediatric	Tocilizumab vs. placebo	Wk 52 mean (95% CI) change in 2 hr MMTT AUC	Placebo: 0.391 (0.47,0.31)<br>Tocilizumab: 0.33 (0.39,0.28), p=0.277

*Studies with significant differences between a treatment group and placebo.
IV – intravenous, MMTT – mixed meal tolerance test, AUC – Area Under the Curve, f/u – follow-up, AbATE - Autoimmunity-Blocking Antibody for Tolerance trial, DEFEND - Durable Response Therapy Evaluation for Early or New-Onset Type 1 Diabetes, HrIFN-α – Human recombinant interferon-alpha, MMF – Mycophenolate mofetil, DZB – Daclizumab, GAD-Alum – Glutamic acid decarboxylase – alum, AIDA – Anti-Interleukin-1 in Diabetes Action, START - Study of Antithymocyte Globulin for Treatment of New-onset T1DM, HCL – Hybrid close Loop, Sc – subcutaneous , ATG – Antithymocyte Globulin, GCSF - Granulocyte Colony-Stimulating Factor, A1AT – Alpha-1 Antitrypsin , T1GER - SIMPONI to Arrest β-cell Loss in Type 1 Diabetes, EXTEND - Tocilizumab (TCZ) in New-onset Type 1 Diabetes

Supplementary Table 3. Papers with Precision Analyses

Study ID	Pre-specified?	Sub-group #	Multiple comp. corrected?	Features used to define subgroups	Smallest sample size	Outcome	Summary
**Prevention**							
Knip 2018[61]	Both	5	No	Age; Sex; Family History of T1D; Specific HLA genotype; Study site/geographic location	2	Time to T1D	No relationships with tx response to extensively hydrolyzed casein formula identified
Näntö-Salonen 2008[62]	Not stated	4	No	Age; Aab #; Specific Aab; β cell function measure	29	Time to T1D	No relationships with intranasal insulin tx response identified
Gale 2004[63]	Pre-specified	5	No	Age; Sex; Aab #; Dysglycemia/AGT; β cell function measure	11	Time to T1D	No relationships with nicotinamide tx response identified
Skyler 2002[64]	Pre-specified	2	No	Dysglycemia/AGT	67	Time to T1D	No relationships with parenteral tx response identified
Skyler 2005[65]	Not stated	1	n/a	Specific Aab	130	Time to T1D	Among participants with higher IAA titer, oral insulin tx associated with reduced risk of progression.
Vehik 2011[66]		1	n/a	Specific Aab	130	Time to T1D	In 75% of original participants with median 9.1 yrs f/u, participants with higher IAA titer maintained tx effect until cessation of therapy, when effect dissipated.
Krischer 2017[67]	Pre-specified	3	No	Aab #; Specific Aab; β cell function measure	55	Time to T1D	IAA+ Participants with ICA+ or GADA and IA2A+ with low FPIR with significant tx response to oral insulin. No significant response in high FPIR group or if ICA+ and GADA or IA2A+.
Elding Larsson 2018[68]	Pre-specified	3	No	Sex; Aab #; Dysglycemia/AGT	7	Time to T1D	No relationships with GAD sc inj tx response identified
Herold 2019[69]	Pre-specified	12	No	Age; Sex; BMI; Specific HLA genotype; Specific	8	Time to T1D	Significant effect of teplizumab vs placebo if: female, BMI >median, GADA+ positive or mIAA+, ICA-, ZnT8A-, or IA2A-, DR3-,

				Aab; β cell function measure; glucose			DR4+, glucose> median, C-peptide AUC< median.
**Prevention Precision**							
Butty 2008[70]	Post-hoc	6	No	Specific HLA genotype, other genetic risk feature	5	Time to T1D	Enhanced effect of oral insulin in those with 1 but not 2 alleles for INS-23A SNP.
Sosenko 2020[71]	Post-hoc	1	n/a	Diabetes progression risk score (DPTRS)	37	C-peptide measure; Time to T1D	For those with DPTRS >=6.75 oral insulin showed significant effect in DPT-1 and in combined data from TN and DPT-1 oral insulin studies.
**New Onset**							
Greenbaum 2021[58]	Both	2	No	Age; genetic risk feature	5	C-peptide measure	No relationships with tocilizumab tx response identified
Wherrett 2011[29]	Both	9	No	Age; Sex; Specific HLA genotype; Specific Aab; β cell function measure; HbA1c	not stated	C-peptide measure	No relationships with GAD sc x treattment response identified
Pescovitz 2009[21]	Not stated	7	No	Age; Sex; Specific HLA genotype; Aab #; β cell function measure; Insulin dose/regimen; HbA1c	11	C-peptide measure	No relationships with rituximab tx response identified
Martin 2011[39]	Not stated	7	No	Age; BMI; Aab #; β cell function measure; Study site/geographic location	n/a	C-peptide measure	No relationships with atorvastatin tx response identified
Lebenthal 2019[51]	Both	2	No	Age; β cell function measure	1 (6% of 20 in placebo group for responder analysis)	C-peptide measure; HbA1c; insulin dose	No relationships with Alpha-1 Antitrypsin tx response identified
Griffin 2014[47]	Pre-specified	5	No	Age; Sex; β cell function measure; Insulin dose or regimen; HbA1c	n/a	C-peptide measure	No relationships with Sitagliptin + lansoprazole tx response identified

Gottlieb 2010[28]	Not stated	6	No	Age; Sex; Aab #;; β cell function measure; Insulin dose/regimen; HbA1c	10	C-peptide measure	No relationships with Mycophenolate mofetil (MMF) +/- Daclizumab (DZB) tx response identified
Chaillous 2000[3]	Not stated	2	No	Age; Measure of β cell function	n/a	C-peptide measure	No relationships with oral human biogenetic insulin tx response identified
Buckingham 2013[46]	Not stated	5	No	Age; Sex; β cell function measure; HbA1c; DKA at dx	4	C-peptide measure	No relationships with hybrid closed loop tx response identified
Aronson 2014[19]	Pre-specified	10	No	Age; Sex; BMI; Aab #; Specific Aab; β cell function measure; Study site/geographic location; Insulin dose or regimen; HbA1c	n/a	C-peptide measure	No relationships with Otelixizumab tx response identified
Rother 2009[27]	Post-hoc	1	n/a	Age	41	C-peptide measure	No relationships with oral interferon alpha tx response identified
Pozzilli 2000[4]	Not stated	1	n/a	Age		C-peptide measure; time to Aab+	No relationships with oral insulin tx response identified
Sherry 2011[13]	Pre-specified	3	No	Age; Study site/geographic location; Duration of dx	31	HbA1c	For 14-day full dose teplizumab, % participants with A1c <7% and lower insulin doses higher in 8-11 year olds, US participants, and participants randomized within 6 wks of dx
Hagopian 2013[72]	Pre-specified	6	No	Age; β cell function measure; Insulin dose/regimen; HbA1c; Study site/geographic location; Duration of dx	31	C-peptide measure	For 14-day full dose teplizumab, 2-yr adjusted mean change in C-peptide AUC showed tx effect in participants in US, randomized in 6 wks from dx, or with baseline A1c<7.5%, insulin dose<0.4 u/kg/day, C-peptide >0.65 or >0.2, or if in the 8-17 yr old age category
Orban 2011[33]	Pre-specified	7	No	Age; Sex; Specific HLA genotype; β cell function measure; Insulin	n/a	C-peptide measure	DR3+ participants with better ratio of abatacept tx effect while nonwhite participants with worse ratio of tx effect.

				dose/regimen; HbA1c; race			
Orban 2014[34]	Pre-specified	8	Yes	Age; Sex; Race; β cell function measure; Insulin dose/regimen; HbA1c; Specific HLA genotype;	3	C-peptide measure	Significant impact of white race and DR3+ status to improve 3-yr C-peptide AUC ratio of tx effect for abatacept vs. placebo, although race effect may be spurious due to small sample size (n=3 in placebo group).
Moran 2013[41]*	Pre-specified	9	No	Age; Sex; BMI; Specific HLA genotype; β cell function measure; Insulin dose or regimen; ethnicity, T1D duration	11	C-peptide measure	Participants with lower tertile of baseline C peptide in the canakinumab-treated group had significantly lower C-peptide concentrations at 1 year
Moran 2013[41]	Pre-specified	9	No	Age; Sex; BMI; Specific HLA genotype; β cell function measure; Insulin dose/regimen; ethnicity, diabetes duration	11	C-peptide measure	No relationships with anakinra tx response identified
Ludvigsson 2012[30]	Pre-specified	13	No	Age; Sex; BMI; Specific HLA genotype; Specific Aab #;; β cell function measure; Study site/geographic location; Insulin dose or regimen; HbA1c; country, days since dx, pubertal stage	n/a	C-peptide measure	Participants who were male (all regimens), had baseline daily insulin dose of 0.398-0.605 IU/kg (all regimens), from non-Nordic European countries (4-dose regimen), or had baseline Tanner pubertal stage of 2 or 3 (4 dose regimen) had higher and significant estimated tx ratios
Herold 2013 [15]	Post-hoc	2	No	Age; HbA1c	7	C-peptide measure	Improved teplizumab tx response in participants who were younger and baseline A1c< 6.5%.
Herold 2013 [16]	Post-hoc	20	No	Age; Sex; BMI; Specific Aab #; β cell function measure; Immune cell phenotype; Insulin dose/regimen; HbA1c	18	C-peptide measure	Clinical responders to teplizumab with lower baseline A1c and insulin use; Baseline CCR4+ naive CCR6+ naive CCR4+ memory CD4+ T cells, CCR4+ naive or IFN-g+ CD8+ T cells higher in

							nonresponders; Baseline effector memory and CD38+ terminally differentiated CD8+ T cells lower in nonresponders.
Gitelman 2021[57]	Post-hoc		No	Age; β cell function measure	n/a	C-peptide measure	Lower baseline Cpeptide AUC associated with better response to imatinib mesylate
Pozzilli [7]1997	Post-hoc	1	n/a	Age	15	C-peptide measure; HbA1c; insulin dose	The insulin dose required to reach the same metabolic control (based on HbA1c) in participants <15 yrs was higher in Vit E-treated vs. nicotinamide-treated participants.
Ludvigsson 2021 [32]	Pre-specified	1	n/a	Specific HLA genotype	19	C-peptide measure	HLA DR3-DQ2+ participants showed greater tx effect of GAD-alum intralymphatic inj + oral vitamin D.
Keymeulen 2005[10]	Not stated	1	n/a	β cell function measure	16	C-peptide measure	Increase in insulin dose over follow up did not occur among participants treated with ChAglyCD3 with higher baseline glucose-clamp C-peptide release (≥P50)
Gitelman 2013[42]	Post-hoc	1	n/a	Age	8	C-peptide measure	No relationships with high dose ATG tx response identified.
Gitelman 2016[43]	Post-hoc	1	n/a	Age	20	C-peptide measure; HbA1c; insulin dose	Older age group (22-35 years) had more "responders: based on C-peptide AUC and showed significant impact of high dose ATG on C-peptide vs. placebo.
Ergun-Longmire 2004[1]	Not stated	1	n/a	Age	n/a	C-peptide measure	Significant benefit of 1 mg and 10mg of oral insulin among subjects >=20 yrs. In patients diagnosed before 20 yrs, 1 mg dose was ineffective, and 10 mg dose accelerated C-peptide loss.
Crinò 2004[5]	Not stated	1	n/a	Age	23	C-peptide measure; HbA1c; insulin dose	For <9 yrs: at 6 months the nicotinamide (NA) +vitamin E group showed significantly higher C-peptide. For >9 yrs: NA alone showed higher C-peptide at 6 months and 9 months.
Coutant 1998[8]	Post-hoc	1	n/a	β cell function measure	40	C-peptide measure;	Linomide tx associated with higher C-peptide in group with >0.1 pmol/L baseline C-peptide.

						HbA1c; insulin dose	
Ataie-Jafari 2013[44]	Not stated	1	n/a	Sex	7	C-peptide measure; daily insulin dose	Males treated with alfacalcidol had improved fasting C-peptide and lower insulin doses by end of study vs. no improvement in females.
Allen 1999[9]	Post-hoc	1	n/a subgroup	Age	34	C-peptide measure	Fasting and stimulated C-peptide lower at all time points in <10 yr group; rate of stimulated C-peptide loss more rapid in <10 yr gr
vonHerrath 2021[53]	Pre-specified	1	n/a	β cell function measure	27	C-peptide measure	Participants with baseline C-peptide > 0.6 nmol/L showed no effect of combination tx with anti-IL-21 and liraglutide.
Walter 2009 [26]	Not stated	3	No	Age; Sex; ethnicity	n/a	C-peptide measure	No relationships with NBI-6024 tx response identified.
Keymeulen 2010 [11]	Post-hoc	2	Yes	Age; β cell function measure	9	C-peptide measure; HbA1c; insulin dose	ChAglyCD3-treated subgroup with initial C-peptide release ≥50th percentile needed lower insulin doses than the corresponding placebo subgroup, but no difference in <50th percentile group; In younger subgroup tx decreased insulin doses and metabolic control vs. placebo at months 24, 36 and 48; In the older subgroup, effect on mean insulin doses only significant and only at 24 months.
**New-onset Precision**							
Christie 2002 [60]	Not stated	5	No	Specific Aab #;	23 for ia2a but numbers not listed for other aabs	C-peptide measure; insulin independence; time in remission	1) Insulin doses lower, stimulated C-peptide was higher, remission rates increased and rate of recurrence lower in cyclosporin treated IA-2A negative group<br><br>2) Cyclosporin tx decreased insulin dose and had a positive effect on stimulated C-peptide in GAD+IA-2A+, GAD-IA-2A-, and

							GAD+IA-2A+ groups, but had minimal effect on the GAD- IA-2A+ group.
Linsley 2019 [37]	Not stated	tested many gene modules	No	Immune cell phenotype; Other: Gene expression modules reflecting immunotypes based on whole blood RNA sequencing	n/a	C-peptide measure	A transient increase in activated B cells, reprogrammed costimulatory ligand gene expression, and reduced inhibition of anti-insulin antibodies immunotype was associated with resistance to abatacept tx; responders to drug were more likely to be older than median age.
Strom 2012 [40]	Not stated	8	No	Age; Sex; BMI; β cell function measure; Immune cell phenotype; Insulin dose or regimen; Total cholesterol and CRP	n/a	C-peptide measure	Lower BMI and higher fasting baseline C-peptide associated with higher median C-peptide in placebo group but not atorvastatin group; Higher CRP in atorvastatin group but not placebo group associated with higher median C-peptide.
Herold 2011 [23]	Not stated	12	No	Immune cell phenotype	19	C-peptide measure	CD3+ and CD4+ cell counts were significantly higher in responders to rituximab. Nonresponders showed no change in proliferative responses to diabetes associated, islet-specific, and neuronal autoantigens over 12 mo.
Demeester 2015 [12]	Not stated	7	No	Age; Specific Aab #; β cell function measure	9	C-peptide measure;	Better otelixizumab response associated with higher titers of mIAA: In the placebo group, patients with higher IAA x C-peptide levels showed rapid loss of functional β cell mass not observed in otelixizumab group.
Cabrera 2018 [36]	Not stated	many transcripts analyzed	Yes	Immune cell phenotype	13	C-peptide measure	Higher baseline inflammatory index in placebo associated with worse C-peptide trajectory but this relationship not present in abatacept tx group, suggesting that higher baseline innate inflammation was associated with better tx response.
Long 2016 [17]	Not stated	Many immune	No	Immune cell phenotype	22	C-peptide measure	A CD8+ T cell population accumulated in teplizumab responders that phenotypically resembled exhausted T cells: expressed

		modules					high levels of the transcription factor EOMES, and multiple inhibitory receptors, including TIGIT and KLRG1.
Long 2017[18]	Not stated	4	Yes	Teplizumab anti-drug antibody (ADA) positivity	5	C-peptide measure; modulation of immune cell phenotype	Only 1/7 teplizumab ADA+ individuals was a clinical responder at 13 mo; ADA+ subjects failed to show CD3 modulation on both CD4+ and CD8+ T cells at the time of the 2nd course. However, after 2nd course, magnitude of CD3 modulation was similar between ADA+ and ADA – groups.
Diggins 2021[59]	Not stated	7	Yes	Immune cell phenotype changes in association with treatment response	6	C-peptide measure	Greater C-peptide preservation by alefacept linked to RNAseq module of CD8+ Tcell activation- and exhaustion-associated genes. Flow cytometry data showed 2 hypoproliferative CD8+ memory cell phenotypes associated with tx response, expressing exhaustion-associated markers TIGIT and KLRG1.
Linsley 2019[24]	Not stated	5	Yes	Immune cell phenotype	n/a	C-peptide measure	Whole blood RNA-seq analysis with flow cytometry f/u testing showed that a transient increase in multiple T cell populations was associated with decreased pharmacodynamic activity of rituximab, increased proliferative response to islet antigens, and rapid C-peptide loss.
Orban 2014[35]	Pre-specified	12	Yes.	Immune cell phenotype	n/a	C-peptide measure; Changes in immune cell subset frequencies	Placebo-treated participants with an increase in central memor CD4 T cells showed subsequent C-peptide decline, but this effect was abrogated by abatacept tx. Abatacept tx resulted in slower C-peptide loss in association with central memory CD4 T cell contraction and naïve CD4 T cell expansion.

Eichmann 2020[38]	Not stated	many subsets	No.	Immune cell phenotype	n/a	C-peptide measure	No relationship with abatacept tx response identified

*Moran 2013 paper includes analyses from 2 trials
Bolding indicates paper classification: prevention, precision prevention, new onset, or precision new onset

Abbreviations: tx- treatment; dx- diagnosis; BMI body mass index; Aab- islet autoantibody; ZnT8- zinc transporter 8 autoantibody; GADA- glutamic acid decarboxylase autantibody; IAA- insulin autoantibody; ICA- islet cell autoantibody; IA2A- islet antigen 2 autoantibody
T1D – type 1 diabetes, HLA - Human Leukocyte Antigens, Tx – treatment, Aab – autoantibody, AGT – Abnormal glucose tolerance, n/a – not applicable, IAA – Insulin autoantibody, f/u – follow-up, ICA – Islet cell autoantibody, GADA – glutamic acid decarboxylase antibody, IA2A – insulinoma-associated protein 2 autoantibody, FPIR – first phase insulin response, Sc – subcutaneous, Inj – injection, BMI – body mass index, ZnT8A - zinc transporter-8 antibody, AUC – area under the curve, SNP – single nucleotide polymorphism, DPTRS – Diabetes Prevention Trial-Type 1 Risk Score, DPT-1 – Diabetes Prevention Trial-Type 1, TN – TrialNet, HbA1c – hemoglobin A1c, GAD Glutamic Acid Decarboxylase, MMF – Mycophenolate Mofetil, DZB – Daclizumab, DKA – Diabetes Ketoacidosis, Dx – diagnosis, ChAglyCD3 – Otelexizumab, ATG - Antithymocyte Globulin, NA – Nicotinamide, Gr – group, CRP – C-reactive protein, ADA – anti-drug antibody

Supplemental References

1 Ergun-Longmire, B. *et al.* Oral insulin therapy to prevent progression of immune-mediated (type 1) diabetes. *Ann N Y Acad Sci* **1029**, 260-277 (2004). https://doi.org:10.1196/annals.1309.057

2 Enander, R. *et al.* Beta cell function after intensive subcutaneous insulin therapy or intravenous insulin infusion at onset of type 1 diabetes in children without ketoacidosis. *Pediatric diabetes* **19**, 1079-1085 (2018). https://doi.org:10.1111/pedi.12657

3 Chaillous, L. *et al.* Oral insulin administration and residual beta-cell function in recent-onset type 1 diabetes: a multicentre randomised controlled trial. Diabète Insuline Orale group. *Lancet* **356**, 545-549 (2000). https://doi.org:10.1016/s0140-6736(00)02579-4

4 Pozzilli, P. *et al.* No effect of oral insulin on residual beta-cell function in recent-onset type I diabetes (the IMDIAB VII). IMDIAB Group. *Diabetologia* **43**, 1000-1004 (2000). https://doi.org:10.1007/s001250051482

5 Crinò, A. *et al.* A randomized trial of nicotinamide and vitamin E in children with recent onset type 1 diabetes (IMDIAB IX). *Eur J Endocrinol* **150**, 719-724 (2004). https://doi.org:10.1530/eje.0.1500719

6 Pitocco, D. *et al.* The effects of calcitriol and nicotinamide on residual pancreatic beta-cell function in patients with recent-onset Type 1 diabetes (IMDIAB XI). *Diabet Med* **23**, 920-923 (2006). https://doi.org:10.1111/j.1464-5491.2006.01921.x

7 Pozzilli, P. *et al.* Vitamin E and nicotinamide have similar effects in maintaining residual beta cell function in recent onset insulin-dependent diabetes (the IMDIAB IV study). *Eur J Endocrinol* **137**, 234-239 (1997). https://doi.org:10.1530/eje.0.1370234

8 Coutant, R. *et al.* Low dose linomide in Type I juvenile diabetes of recent onset: a randomised placebo-controlled double blind trial. *Diabetologia* **41**, 1040-1046 (1998). https://doi.org:10.1007/s001250051028

9 Allen, H. F. *et al.* Effect of Bacillus Calmette-Guerin vaccination on new-onset type 1 diabetes. A randomized clinical study. *Diabetes Care* **22**, 1703-1707 (1999). https://doi.org:10.2337/diacare.22.10.1703

10 Keymeulen, B. *et al.* Insulin needs after CD3-antibody therapy in new-onset type 1 diabetes. *N Engl J Med* **352**, 2598-2608 (2005). https://doi.org:10.1056/NEJMoa043980

11 Keymeulen, B. *et al.* Four-year metabolic outcome of a randomised controlled CD3-antibody trial in recent-onset type 1 diabetic patients depends on their age and baseline residual beta cell mass. *Diabetologia* **53**, 614-623 (2010). https://doi.org:10.1007/s00125-009-1644-9

12 Demeester, S. *et al.* Preexisting insulin autoantibodies predict efficacy of otelixizumab in preserving residual β-cell function in recent-onset type 1 diabetes. *Diabetes Care* **38**, 644-651 (2015). https://doi.org:10.2337/dc14-1575

13 Sherry, N. *et al.* Teplizumab for treatment of type 1 diabetes (Protégé study): 1-year results from a randomised, placebo-controlled trial. *Lancet* **378**, 487-497 (2011). https://doi.org:10.1016/s0140-6736(11)60931-8

14 Hagopian, W. *et al.* Teplizumab preserves C-peptide in recent-onset type 1 diabetes: two-year results from the randomized, placebo-controlled Protégé trial. *Diabetes* **62**, 3901-3908 (2013). https://doi.org:10.2337/db13-0236
15 Herold, K. C. *et al.* Teplizumab treatment may improve C-peptide responses in participants with type 1 diabetes after the new-onset period: a randomised controlled trial. *Diabetologia* **56**, 391-400 (2013). https://doi.org:10.1007/s00125-012-2753-4
16 Herold, K. C. *et al.* Teplizumab (anti-CD3 mAb) treatment preserves C-peptide responses in patients with new-onset type 1 diabetes in a randomized controlled trial: metabolic and immunologic features at baseline identify a subgroup of responders. *Diabetes* **62**, 3766-3774 (2013). https://doi.org:10.2337/db13-0345
17 Long, S. A. *et al.* Partial exhaustion of CD8 T cells and clinical response to teplizumab in new-onset type 1 diabetes. *Sci Immunol* **1** (2016). https://doi.org:10.1126/sciimmunol.aai7793
18 Long, S. A. *et al.* Remodeling T cell compartments during anti-CD3 immunotherapy of type 1 diabetes. *Cell Immunol* **319**, 3-9 (2017). https://doi.org:10.1016/j.cellimm.2017.07.007
19 Aronson, R. *et al.* Low-dose otelixizumab anti-CD3 monoclonal antibody DEFEND-1 study: results of the randomized phase III study in recent-onset human type 1 diabetes. *Diabetes Care* **37**, 2746-2754 (2014). https://doi.org:10.2337/dc13-0327
20 Ambery, P. *et al.* Efficacy and safety of low-dose otelixizumab anti-CD3 monoclonal antibody in preserving C-peptide secretion in adolescent type 1 diabetes: DEFEND-2, a randomized, placebo-controlled, double-blind, multi-centre study. *Diabet Med* **31**, 399-402 (2014). https://doi.org:10.1111/dme.12361
21 Pescovitz, M. D. *et al.* Rituximab, B-lymphocyte depletion, and preservation of beta-cell function. *N Engl J Med* **361**, 2143-2152 (2009). https://doi.org:10.1056/NEJMoa0904452
22 Pescovitz, M. D. *et al.* B-lymphocyte depletion with rituximab and β-cell function: two-year results. *Diabetes Care* **37**, 453-459 (2014). https://doi.org:10.2337/dc13-0626
23 Herold, K. C. *et al.* Increased T cell proliferative responses to islet antigens identify clinical responders to anti-CD20 monoclonal antibody (rituximab) therapy in type 1 diabetes. *J Immunol* **187**, 1998-2005 (2011). https://doi.org:10.4049/jimmunol.1100539
24 Linsley, P. S. *et al.* Elevated T cell levels in peripheral blood predict poor clinical response following rituximab treatment in new-onset type 1 diabetes. *Genes Immun* **20**, 293-307 (2019). https://doi.org:10.1038/s41435-018-0032-1
25 Ortqvist, E. *et al.* Temporary preservation of beta-cell function by diazoxide treatment in childhood type 1 diabetes. *Diabetes Care* **27**, 2191-2197 (2004). https://doi.org:10.2337/diacare.27.9.2191
26 Walter, M., Philotheou, A., Bonnici, F., Ziegler, A. G. & Jimenez, R. No effect of the altered peptide ligand NBI-6024 on beta-cell residual function and insulin needs in new-onset type 1 diabetes. *Diabetes Care* **32**, 2036-2040 (2009). https://doi.org:10.2337/dc09-0449

27 Rother, K. I. *et al.* Effect of ingested interferon-alpha on beta-cell function in children with new-onset type 1 diabetes. *Diabetes Care* **32**, 1250-1255 (2009). https://doi.org:10.2337/dc08-2029

28 Gottlieb, P. A. *et al.* Failure to preserve beta-cell function with mycophenolate mofetil and daclizumab combined therapy in patients with new- onset type 1 diabetes. *Diabetes Care* **33**, 826-832 (2010). https://doi.org:10.2337/dc09-1349

29 Wherrett, D. K. *et al.* Antigen-based therapy with glutamic acid decarboxylase (GAD) vaccine in patients with recent-onset type 1 diabetes: a randomised double-blind trial. *Lancet* **378**, 319-327 (2011). https://doi.org:10.1016/s0140-6736(11)60895-7

30 Ludvigsson, J. *et al.* GAD65 antigen therapy in recently diagnosed type 1 diabetes mellitus. *N Engl J Med* **366**, 433-442 (2012). https://doi.org:10.1056/NEJMoa1107096

31 Ludvigsson, J. *et al.* Combined vitamin D, ibuprofen and glutamic acid decarboxylase-alum treatment in recent onset Type i diabetes: Lessons from the DIABGAD randomized pilot trial. *Future Science OA* **6** (2020). https://doi.org:10.2144/fsoa-2020-0078

32 Ludvigsson, J. *et al.* Intralymphatic Glutamic Acid Decarboxylase With Vitamin D Supplementation in Recent-Onset Type 1 Diabetes: A Double-Blind, Randomized, Placebo-Controlled Phase IIb Trial. *Diabetes Care* **44**, 1604-1612 (2021). https://doi.org:10.2337/dc21-0318

33 Orban, T. *et al.* Co-stimulation modulation with abatacept in patients with recent-onset type 1 diabetes: a randomised, double-blind, placebo-controlled trial. *Lancet* **378**, 412-419 (2011). https://doi.org:10.1016/s0140-6736(11)60886-6

34 Orban, T. *et al.* Costimulation modulation with abatacept in patients with recent-onset type 1 diabetes: follow-up 1 year after cessation of treatment. *Diabetes Care* **37**, 1069-1075 (2014). https://doi.org:10.2337/dc13-0604

35 Orban, T. *et al.* Reduction in CD4 central memory T-cell subset in costimulation modulator abatacept-treated patients with recent-onset type 1 diabetes is associated with slower C-peptide decline. *Diabetes* **63**, 3449-3457 (2014). https://doi.org:10.2337/db14-0047

36 Cabrera, S. M. *et al.* Innate immune activity as a predictor of persistent insulin secretion and association with responsiveness to CTLA4-Ig treatment in recent-onset type 1 diabetes. *Diabetologia* **61**, 2356-2370 (2018). https://doi.org:10.1007/s00125-018-4708-x

37 Linsley, P. S., Greenbaum, C. J., Speake, C., Long, S. A. & Dufort, M. J. B lymphocyte alterations accompany abatacept resistance in new-onset type 1 diabetes. *JCI Insight* **4** (2019). https://doi.org:10.1172/jci.insight.126136

38 Eichmann, M. *et al.* Costimulation Blockade Disrupts CD4(+) T Cell Memory Pathways and Uncouples Their Link to Decline in beta-Cell Function in Type 1 Diabetes. *J Immunol* **204**, 3129-3138 (2020). https://doi.org:10.4049/jimmunol.1901439

39 Martin, S. *et al.* Residual beta cell function in newly diagnosed type 1 diabetes after treatment with atorvastatin: the Randomized DIATOR Trial. *PLoS One* **6**, e17554 (2011). https://doi.org:10.1371/journal.pone.0017554

40 Strom, A. *et al.* Improved preservation of residual beta cell function by atorvastatin in patients with recent onset type 1 diabetes and high CRP levels (DIATOR trial). *PLoS One* **7**, e33108 (2012). https://doi.org:10.1371/journal.pone.0033108
41 Moran, A. *et al.* Interleukin-1 antagonism in type 1 diabetes of recent onset: two multicentre, randomised, double-blind, placebo-controlled trials. *Lancet* **381**, 1905-1915 (2013). https://doi.org:10.1016/s0140-6736(13)60023-9
42 Gitelman, S. E. *et al.* Antithymocyte globulin treatment for patients with recent-onset type 1 diabetes: 12-month results of a randomised, placebo-controlled, phase 2 trial. *Lancet Diabetes Endocrinol* **1**, 306-316 (2013). https://doi.org:10.1016/s2213-8587(13)70065-2
43 Gitelman, S. E. *et al.* Antithymocyte globulin therapy for patients with recent-onset type 1 diabetes: 2 year results of a randomised trial. *Diabetologia* **59**, 1153-1161 (2016). https://doi.org:10.1007/s00125-016-3917-4
44 Ataie-Jafari, A. *et al.* A randomized placebo-controlled trial of alphacalcidol on the preservation of beta cell function in children with recent onset type 1 diabetes. *Clin Nutr* **32**, 911-917 (2013). https://doi.org:10.1016/j.clnu.2013.01.012
45 Nafei, L. T., Kadhim, K. A., Said, A. M. & Ghani, S. H. Evaluation the effect of vitamin D3 on glycemic indices on Iraqi children with type 1 DM. *International Journal of Pharmaceutical Sciences Review and Research* **42**, 134-143 (2017).
46 Buckingham, B. *et al.* Effectiveness of early intensive therapy on β-cell preservation in type 1 diabetes. *Diabetes Care* **36**, 4030-4035 (2013). https://doi.org:10.2337/dc13-1074
47 Griffin, K. J., Thompson, P. A., Gottschalk, M., Kyllo, J. H. & Rabinovitch, A. Combination therapy with sitagliptin and lansoprazole in patients with recent-onset type 1 diabetes (REPAIR-T1D): 12-month results of a multicentre, randomised, placebo-controlled, phase 2 trial. *Lancet Diabetes Endocrinol* **2**, 710-718 (2014). https://doi.org:10.1016/s2213-8587(14)70115-9
48 Pozzilli, P. *et al.* Randomized 52-week Phase 2 Trial of Albiglutide Versus Placebo in Adult Patients With Newly Diagnosed Type 1 Diabetes. *J Clin Endocrinol Metab* **105** (2020). https://doi.org:10.1210/clinem/dgaa149
49 Haller, M. J. *et al.* Low-Dose Anti-Thymocyte Globulin (ATG) Preserves β-Cell Function and Improves HbA(1c) in New-Onset Type 1 Diabetes. *Diabetes Care* **41**, 1917-1925 (2018). https://doi.org:10.2337/dc18-0494
50 Haller, M. J. *et al.* Low-Dose Anti-Thymocyte Globulin Preserves C-Peptide, Reduces HbA(1c), and Increases Regulatory to Conventional T-Cell Ratios in New-Onset Type 1 Diabetes: Two-Year Clinical Trial Data. *Diabetes* **68**, 1267-1276 (2019). https://doi.org:10.2337/db19-0057
51 Lebenthal, Y. *et al.* A Phase II, Double-Blind, Randomized, Placebo-Controlled, Multicenter Study Evaluating the Efficacy and Safety of Alpha-1 Antitrypsin (AAT) (Glassia(®)) in the Treatment of Recent-Onset Type 1 Diabetes. *Int J Mol Sci* **20** (2019). https://doi.org:10.3390/ijms20236032
52 Quattrin, T. *et al.* Golimumab and Beta-Cell Function in Youth with New-Onset Type 1 Diabetes. *N Engl J Med* **383**, 2007-2017 (2020). https://doi.org:10.1056/NEJMoa2006136

53 von Herrath, M. *et al.* Anti-interleukin-21 antibody and liraglutide for the preservation of β-cell function in adults with recent-onset type 1 diabetes: a randomised, double-blind, placebo-controlled, phase 2 trial. *Lancet Diabetes Endocrinol* **9**, 212-224 (2021). https://doi.org:10.1016/s2213-8587(21)00019-x
54 Lagarde, W. H. *et al.* Human plasma-derived alpha(1) -proteinase inhibitor in patients with new-onset type 1 diabetes mellitus: A randomized, placebo-controlled proof-of-concept study. *Pediatr Diabetes* **22**, 192-201 (2021). https://doi.org:10.1111/pedi.13162
55 Kumar, S. *et al.* A high potency multi-strain probiotic improves glycemic control in children with new-onset type 1 diabetes mellitus: A randomized, double-blind, and placebo-controlled pilot study. *Pediatric diabetes* **22**, 1014-1022 (2021). https://doi.org:10.1111/pedi.13244
56 Groele, L. *et al.* Lack of effect of Lactobacillus rhamnosus GG and Bifidobacterium lactis Bb12 on beta-cell function in children with newly diagnosed type 1 diabetes: a randomised controlled trial. *BMJ Open Diabetes Res Care* **9** (2021). https://doi.org:10.1136/bmjdrc-2020-001523
57 Gitelman, S. E. *et al.* Imatinib therapy for patients with recent-onset type 1 diabetes: a multicentre, randomised, double-blind, placebo-controlled, phase 2 trial. *Lancet Diabetes Endocrinol* **9**, 502-514 (2021). https://doi.org:10.1016/s2213-8587(21)00139-x
58 Greenbaum, C. J. *et al.* IL-6 receptor blockade does not slow beta cell loss in new-onset type 1 diabetes. *JCI Insight* **6** (2021). https://doi.org:10.1172/jci.insight.150074
59 Diggins, K. E. *et al.* Exhausted-like CD8+ T cell phenotypes linked to C-peptide preservation in alefacept-treated T1D subjects. *JCI Insight* **6** (2021). https://doi.org:10.1172/jci.insight.142680
60 Christie, M. R., Mølvig, J., Hawkes, C. J., Carstensen, B. & Mandrup-Poulsen, T. IA-2 antibody-negative status predicts remission and recovery of C-peptide levels in type 1 diabetic patients treated with cyclosporin. *Diabetes Care* **25**, 1192-1197 (2002). https://doi.org:10.2337/diacare.25.7.1192
61 Knip, M. *et al.* Effect of Hydrolyzed Infant Formula vs Conventional Formula on Risk of Type 1 Diabetes. *JAMA* **319**, 38 (2018). https://doi.org:10.1001/jama.2017.19826
62 Näntö-Salonen, K. *et al.* Nasal insulin to prevent type 1 diabetes in children with HLA genotypes and autoantibodies conferring increased risk of disease: a double-blind, randomised controlled trial. *Lancet* **372**, 1746-1755 (2008). https://doi.org:10.1016/s0140-6736(08)61309-4
63 Gale, E. A., Bingley, P. J., Emmett, C. L. & Collier, T. European Nicotinamide Diabetes Intervention Trial (ENDIT): a randomised controlled trial of intervention before the onset of type 1 diabetes. *Lancet* **363**, 925-931 (2004). https://doi.org:10.1016/s0140-6736(04)15786-3
64 Effects of insulin in relatives of patients with type 1 diabetes mellitus. *N Engl J Med* **346**, 1685-1691 (2002). https://doi.org:10.1056/NEJMoa012350
65 Skyler, J. S. *et al.* Effects of oral insulin in relatives of patients with type 1 diabetes: The Diabetes Prevention Trial--Type 1. *Diabetes Care* **28**, 1068-1076 (2005). https://doi.org:10.2337/diacare.28.5.1068

66 Vehik, K. *et al.* Long-term outcome of individuals treated with oral insulin: diabetes prevention trial-type 1 (DPT-1) oral insulin trial. *Diabetes Care* **34**, 1585-1590 (2011). https://doi.org:10.2337/dc11-0523
67 Krischer, J. P., Schatz, D. A., Bundy, B., Skyler, J. S. & Greenbaum, C. J. Effect of Oral Insulin on Prevention of Diabetes in Relatives of Patients With Type 1 Diabetes: A Randomized Clinical Trial. *Jama* **318**, 1891-1902 (2017). https://doi.org:10.1001/jama.2017.17070
68 Elding Larsson, H., Lundgren, M., Jonsdottir, B., Cuthbertson, D. & Krischer, J. Safety and efficacy of autoantigen-specific therapy with 2 doses of alum-formulated glutamate decarboxylase in children with multiple islet autoantibodies and risk for type 1 diabetes: A randomized clinical trial. *Pediatr Diabetes* **19**, 410-419 (2018). https://doi.org:10.1111/pedi.12611
69 Herold, K. C. *et al.* An Anti-CD3 Antibody, Teplizumab, in Relatives at Risk for Type 1 Diabetes. *New England Journal of Medicine* **381**, 603-613 (2019). https://doi.org:10.1056/nejmoa1902226
70 Butty, V., Campbell, C., Mathis, D. & Benoist, C. Impact of diabetes susceptibility loci on progression from pre-diabetes to diabetes in at-risk individuals of the diabetes prevention trial-type 1 (DPT-1). *Diabetes* **57**, 2348-2359 (2008). https://doi.org:10.2337/db07-1736
71 Sosenko, J. M. *et al.* Slowed Metabolic Decline After 1 Year of Oral Insulin Treatment Among Individuals at High Risk for Type 1 Diabetes in the Diabetes Prevention Trial-Type 1 (DPT-1) and TrialNet Oral Insulin Prevention Trials. *Diabetes* **69**, 1827-1832 (2020). https://doi.org:10.2337/db20-0166
72 Hagopian, W. *et al.* Teplizumab preserves C-peptide in recent-onset type 1 diabetes: two-year results from the randomized, placebo-controlled Protege trial. *Diabetes* **62**, 3901-3908 (2013). https://doi.org:10.2337/db13-0236
