# Supplementary material for: Disease-modifying therapies and features linked to treatment response in type 1 diabetes prevention: a systematic review
Source: Commun Med (Lond). 2023 Oct 5;3:130. doi: 10.1038/s43856-023-00357-y (PMC10550983; doi:10.1038/s43856-023-00357-y)
Supplement: Supplementary file 4 — Description of Additional Supplementary Files [file 43856_2023_357_MOESM4_ESM.pdf]

Description of Additional Supplementary Files

File Name: Supplementary Data 1

Description: Supplementary Tables in Excel format

File Name: Supplementary Data 2

Description: Raw data plotted in figures 2-5
